# Bartholinitis due to *Aggregatibacter aphrophilus*: a case report

**DOI:** 10.1186/s12879-016-1908-1

**Published:** 2016-10-18

**Authors:** Morgane Choquet, Emilie Pluquet, Sandrine Castelain, Raphaël Guihéneuf, Véronique Decroix

**Affiliations:** 1 Department of Bacteriology, Amiens University Hospital, Amiens, France; 2 Microbiology Research Unit, EA4294, Jules Verne University of Picardie, Amiens, France

**Keywords:** *Aggregatibacter aphrophilus*, Bartholinitis, Gram negative coccobacilli, Capnophilic bacteria, Genital infections

## Abstract

**Background:**

*Aggregatibacter aphrophilus,* a commensal of the oro-pharyngeal flora and member of the HACEK group of organisms, is an uncommonly encountered clinical pathogen. It has already been described as the causative agent of brain abscesses, empyema, meningitis, sinusitis, otitis media, bacteriemia, pneumonia, osteomyelitis, peritonitis, endocarditis and wound infections. Herein we report the first case of bartholinitis due to *A. aphrophilus.*

**Case presentation:**

A 33-year-old woman was admitted for a 3-day genital pain without fever and urinary functional signs. The abscess was incised and drained; *A. aphrophilus* was the only micro-organism that grew from the pus. The patient received no antibiotics; the clinical course was favourable.

**Conclusion:**

This case highlights the importance of an effective treatment of recurrent bartholinitis such as a cold resection of the gland. It is presented for its rarity.

## Background

*Aggregatibacter aphrophilus* is a Gram negative, oxidase and catalase negative, capnophilic, fermentative coccobacillus with X independence on primary isolation [1]. It is a member of the HACEK group of organisms (*Haemophilus, Aggregatibacter, Cardiobacterium, Eikenella and Kingella*) [2] and was formerly named *Haemophilus aphrophilus* or *Haemophilus paraphrophilus*.

*A. aphrophilus* is part of the normal oropharyngeal flora and has already been described as the causative agent of brain abscesses, empyema, meningitis, sinusitis, otitis media, bacteriemia, pneumonia, osteomyelitis, peritonitis, endocarditis and wound infections [1].

The Bartholin’s glands are two little hormonodependent glands that are located symmetrically in the posterior region of the vaginal opening and that take part in mucus secretion and vaginal lubrification. Acute bartholinitis is generally due to the surinfection of a gland cyst caused by the obstruction of the excretory duct. The main clinical sign is a painful swelling in the posterior area of the labia majora [3].

Most cases of bartholinitis have been thought to be caused by micro-organisms of the vaginal flora (such as Enterobacteriaceae, *Enterococcus spp*. or anaerobic bacteria). *Neisseria gonorrhoeae* and *Chlamydia trachomatis* are of limited aetiologic importance as causes of Bartholin’s duct abscess [4, 5].

We will report a case of bartholinitis due to *A. aphrophilus,* an organism not previously described in the literature as causing such infections.

## Case presentation

A 33-year-old non pregnant woman (G3P1) was admitted to the obstetric emergency ward of Amiens Teaching Hospital (Amiens, France) for a 3-day genital pain located in the left labia majora. Her past medical history was unremarkable (term delivery, normal blood sugar, no previous history of diabetes in her family) except for a recurrent left Bartholin’s gland abscess. On admission, she was afebrile and presented no urinary functional signs. Gynecologic examination revealed a large abscess which was incised and drained under spinal anesthesia. The purulent fluid was sent for culture to the bacteriology laboratory.

**Fig. 1:**
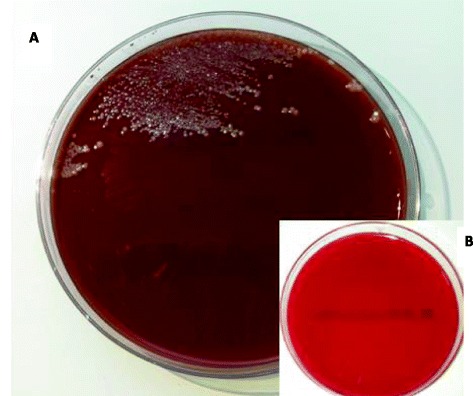
Culture of *A. aphrophilus* on chocolate agar (**a**) and blood agar (**b**) plates after 48 h under 5 % CO2

Gram staining of the pus showed a lot of neutrophils with Gram negative coccobacilli and the next day cultures revealed a pure culture of small, high-convex, grey colonies only growing on chocolate culture medium (Fig. 1) (Polyvitex PVX, bioMérieux, France) under 5 % CO_2_. Catalase and oxidase reactions were negative. The isolate was identified with the use of matrix-assisted laser desorption ionisation-time of flight mass spectrometry (MALDI-TOF MS; Bruker Daltonik GmbH, Germany; MALDI Biotyper 2.2) as *A. aphrophilus*. Identification was confirmed by sequencing the 16S RNA gene as previously described [6]. The isolate was identified as *A. aphrophilus* with a maximum identity of 100 % for *A. aphrophilus* strain NJ8700 (GenBank accession number CP009230.1). Susceptibility testing was performed using E-test (bioMérieux, France) on chocolate culture medium (Polyvitex PVX, bioMérieux, France) under 5 % CO_2_. The minimal inhibitory concentrations (MICs) for amoxicillin, amoxicillin-clavulanic acid, ceftriaxone, tetracycline and rifampicin were 0.38 mg/L, 0.38 mg/L, < 0.016 mg/L, 1.0 mg/L and 0.25 mg/L respectively.

The patient received no antibiotics for the treatment of this left bartholinitis and the clinical course was favourable. She went home a few days later with only local care and analgesics. A cold resection was recommended by the surgeon who operated on her to prevent any recurrence but could not have been done.

Unfortunately, the woman came back to the hospital 5 months later for a new abscess of the Bartholin’s left gland. A wild-type *Escherichia coli* was isolated and the treatment was the same as for the previous abscess, except that a marsupialization was performed.

## Discussion

*A. aphrophilus* was first described by Khairat [7] in 1940 (initially as *Haemophilus aphrophilus*) after it had been isolated from a patient with infectious endocarditis. This bacterium causes many different kinds of infections (more or less severe) but has never been documented as the causative agent of bartholinitis.

Bartholinitis is quite a common problem in clinical practice and can be caused by a wide variety of micro-organisms [3]. First, Enterobacteriaceae (mainly *E. coli*) followed by capnophilic species such as *Haemophilus influenzae* and *N. gonorrhoeae* [8]. Anaerobic bacteria from the vaginal flora have also been implicated, mostly in polymicrobial abscesses due to both aerobes and anaerobes [5]. *C. trachomatis* should be considered as a rare cause of bartholinitis.

Nulligravida women between 20 and 29 years old and patients with diabetes are the most affected populations [9]. In our case, the aetiology couldn’t be found. Indeed, the patient was 33 years old, she had a son, her blood sugar was normal (4.4 mmol/L with an empty stomach) and there was no previous history of diabetes in her family.

Following bartholinitis, haemorrhagic or infectious complications can occur such as septicaemia, pelviperineal abscesses or necrotizing fasciitis, which remains a rare phenomenon. The recurrence rate has been evaluated between 5 and 15 % after a first episode and this rate is not modified by marsupialisation [10]. Concerning our case, it was the third episode of bartholinitis (previously a *Streptococcus anginosus* had been isolated; and for the second one, no bacteriological sample had been collected) and 5 months later, the patient presented a new recurrence.

It has been suggested that the change in sexual practices modifies the ecology of Bartholin’s glands abscesses [11]. While a diminution of infections due to bacteria involved in sexually transmitted diseases can be observed nowadays, abscesses caused by micro-organisms from the gastro-intestinal flora, but also from the oro-pharyngeal flora are mostly described. For example, *Streptococcus pneumoniae*, a commensal of the upper respiratory tract, can be responsible for such infections as a result of orogenital contact [3, 11]. Another case of Bartholin’s gland abscess caused by a micro-organism from the oro-pharyngeal flora, *Neisseria sicca*, was reported in the literature by Berger et al. in 1988 [12].

The recommended treatment for a Bartholin’s gland abscess is surgical incision and draining [5]. However, the introduction of antibiotics for the treatment of such infections is much discussed. Three opinions emerge from the literature. First, antibiotics introduced as a complement to surgery are advised (an intraoperative intravenous injection) although long term antibiotherapy is considered useless. Secondly, no other treatment than marsupialization is advocated. Finally, antibiotic treatment can be considered in a context of pregnancy [5]. Our patient received no antibiotics for any of her four episodes of bartholinitis.

## Conclusion

An overview of the literature makes us note that *A. aphrophilus* has never been documented in acute bartholinitis. However, this organism, part of the oro-pharyngeal flora, has already been described as the causative agent of many different kinds of infections.

Recurrent bartholinitis in our case highlights the importance of an effective treatment of this type of infection.
